# Coping Ability and Promotion Countermeasures of Medical and Health Institutions Reputation Crisis: A Case Study in Hubei Province

**DOI:** 10.3389/fpubh.2021.802004

**Published:** 2022-02-09

**Authors:** Yang Liu, Xiaofang Li, Rui Ding, Tianrun He, Xian-jia Wang

**Affiliations:** ^1^Zhongnan Hospital of Wuhan University, Wuhan University, Wuhan, China; ^2^School of Economics and Management, Wuhan University, Wuhan, China; ^3^School of Journalism and Communication, Wuhan University, Wuhan, China; ^4^Hubei Media Group, Wuhan, China

**Keywords:** coping ability, promotion countermeasures, situational crisis communication theory, medical and health institutions, reputation crisis

## Abstract

At present, the doctor–patient relationships in Chinese medical and health institutions (MHI) are increasingly tense, and the reputation crisis challenges are being faced by MHI more frequently. However, the reputation crisis management level of the MHI is directly related to the future development and construction of the MHI and is an important management link that cannot be ignored. Therefore, how to quantify the impact of the relevant crisis on hospitals has become a major problem. First of all, this paper uses the situational crisis communication theory (SCCT) to combine the characteristics of hospital reputation crisis with the theory and classification of MHI reputation crisis from three perspectives: victim crisis, accidental crisis, and preventable crisis. Second, a more comprehensive analysis of MHI reputation crisis management model is conducted in the research cases, based on the relevant data of Union Hospital, Tongji Medical College Huazhong University of Science and Technology (UH), Tongji Hospital, Tongji Medical College Huazhong University of Science and Technology (TJ), Zhongnan Hospital of Wuhan University (ZN), Renmin Hospital of Wuhan University (RM), and Maternal and Child Hospital of Hubei Province (MC). Third, we divide MHI reputation positioning into four types, namely robust, growth, fragile, and sensitive, and innovate SCCT to build a new MHI crisis classification type. Finally, this paper provides appropriate crisis management strategies for sample MHI based on the above examples and theories. Furthermore, we realize the lifecycle management of MHI reputation by identifying, evaluating and responding to reputation issues. This study provides a theoretical reference for the MHI reputation crisis management level and the adjustment of future management strategies.

## Introduction

Medical and Health Institution (MHI) reputation is the rational cognition and emotional evaluation of the past and current behavior and results of a MHI through personal experience or through internet and word-of-mouth communication. For the 2019 China Hospital Rankings that were released by the Hospital Management Institute of Fudan University (1), the total score of each MHI is calculated based on two factors: reputation and scientific research. Peking Union Medical College Hospital has been ranked first for 10 consecutive years. This ranking has become a reference standard and guideline for major MHI and patients in China. Patients' trust in a MHI is based on the MHI reputation, and patients also prefer to go to general MHI with higher average reputation values for medical treatment (2). With the advancement of the reform of the medical system, China's medical and health services have undergone rapid development, and public MHI also occupy an important position in patient care and social development (3). Among them, doctor–patient relationship is a sensitive point in the medical field, and various medical disputes and medical incidents have greatly affected and impacted the doctor–patient relationship, which is the main part of the MHI reputation management (4–6). Research on MHI reputation and quantitative monitoring and evaluation can visually display the key elements of MHI reputation, which is essential for accurate management of MHI reputation (7, 8). Taking UH, TJ, ZN, RM, and MC as the research samples, this study establishes the MHI reputation crisis management index model and uses the situational crisis communication theory (SCCT) to explain the response strategies to the MHI reputation crisis (9). This paper makes innovations based on the SCCT and adopts a more subdivided crisis classification, and a relatively modest response to the crisis.

## Medical and Health Institutions Reputation Definition

Nowadays, there are mainly two perspectives on reputation research. One is from the perspective of economics, which regards reputation as an important mechanism to ensure the honest execution of contracts (10). The other is a management perspective, which treats reputation as an intangible resource and asset (11). Although there is no uniform definition of corporate reputation, it mainly has the following characteristics (12). One is the overall evaluation and impression of many aspects of the company. The second is that the evaluator of corporate reputation can be a single subject or multiple subjects. The third is that the source of corporate reputation is all past behaviors of the company. Fourth, the relevant entities evaluate the corporate reputation based on direct experience and indirect information. Fifth, the core of corporate reputation is trust.

MHI reputation refers to the reputation and reputation of the MHI in the public, which is an intangible asset and potential and important resource. MHI reputation management is the development and utilization of such intangible resources (13). The MHI is born with the public welfare nature of rescuing the dying. Although the MHI reputation is based on the reputation and enterprise reputation research, it has its special attributes, which is different from ordinary enterprises with profit as the first goal (14).

The existing research on MHI reputation is mainly reflected in the discussion of MHI reputation definition and reputation management. Asenjo et al. (15) proposed a method to analyze the reputation parameters of Spanish MHI, which shows that scientific production and reputation index are positively correlated. Triemstra et al. (7) found that MHI reputation may be affected by its social media presence, or the MHI reputation or ranking may drive social media followers. Ziemba et al. (16) believed that consumers associate the construction of MHI reputation with objective medical quality. This behavioral pattern is worthy of attention, especially when the reputation is inconsistent with objective data. MHI is a kind of non-profit organization with the characteristics of public welfare, productivity, and operation. It integrates the definition of corporate reputation and the characteristics of MHI. Specifically, MHI reputation refers to what people perceive based on direct experience and indirect information. The overall impression and evaluation of a MHI is a comprehensive reflection of all aspects of a MHI's behavioral abilities, formed in the long-term conscious medical service process (17). The research perspective of corporate reputation generally considers the customer as God as the basic purpose and defines consumers as rational economic people, which fits the first goal of the enterprise profitability. However, when studying MHI reputation, both patients and doctors are irrational economic people. The goal is not to obtain cost-effective products, but to obtain treatment and treatment for diseases. Therefore, MHI reputation is not a purely economic concept, but a balance between social and economic benefits. The reputation of MHI cannot be measured with complete profitability.

### SCCT and Application

The SCCT was proposed by Coombs (18). To quantify the effect of crisis response, the SCCT was tested by testing the audience's perception and attitude after communication, various communication strategy adaptation conditions were summarized, and then the SCCT was proposed. SCCT mainly includes crisis scenarios, crisis response strategies, and crisis scenario adaptation systems. Crisis scenarios include three dimensions: crisis responsibility, organizational crisis history, and past reputation. At the same time, Coombs introduced the attribution theory in psychology, emphasizing that crisis classification should be judged based on the subjective perception of the public, that is to say, if stakeholders believe that the organization is guilty, then the organization has an organizational crisis. He also pointed out that anger and compassion are the two major emotional factors that affect people's attribution. According to the stakeholder's attribution of organizational responsibility, the types of crises are divided into three types: victimized, accidental, and preventable.

After the crisis occurs, the organization's primary task is to prevent stakeholders from harming. Therefore, organizations must provide indicative information after a crisis to prevent people from being physically harmed and provide adaptive information to deal with people's psychological threats. Based on this, the organization can adopt the four basic crisis communication strategies: denial, downplay, reconstruction, and support. The key to matching crisis scenarios and crisis response strategies lies in their respective strengths and weaknesses. On the one hand, the attribution of responsibility becomes stronger from the victim type, accident type, and preventable crisis type. On the other hand, the tough attitude of denial, downplay, and reconstruction strategy weakens in turn. In this way, the two form a dynamic adaptation system.

MHI reputation crisis management can refer to the classification of crisis in SCCT, list the common crisis scenarios according to the actual situation of MHI reputation, and adopt targeted crisis response strategies. That is, SCCT believes that organizations first classify the crisis when a crisis occurs and choose the most appropriate crisis response strategy after evaluating the variables that may affect the enterprise reputation. That is to say, different crisis communication strategies have different effects. The key to achieve the best communication effect is in the adaptation of communication strategies and crisis scenarios and to choose the corresponding strategies according to the size of responsibility of the organization. Specifically, when the organization (MHI) is a unresponsible victim crisis scenario, denial strategy can be adopted; when the organization (MHI) is a moderately responsible accidental crisis situation, excuse, rationalization, and other elimination strategies can be adopted; when the organization (MHI) is a highly responsible misbehavior crisis situation, the organization should not escape, should actively assume responsibility, and should adopt communication strategies such as correction and apology.

According to the data analysis of this article, there are no major crisis events occurred in the five MHI in Hubei Province, mainly some smaller public opinion events. Therefore, the corresponding treatment of the response strategy will be unsuitable and needs to be adjusted accordingly. Then the strategy has been adjusted and corrected accordingly, and low victim crisis risk can be applicable to resistant management response strategy. High crisis risk can be prevented, and a gentle defensive strategy should be used.

## Construction and Analysis of MHI Reputation Crisis Management Index Model

### Evaluation Index System of MHI Reputation Crisis Management

**Figure 1:**
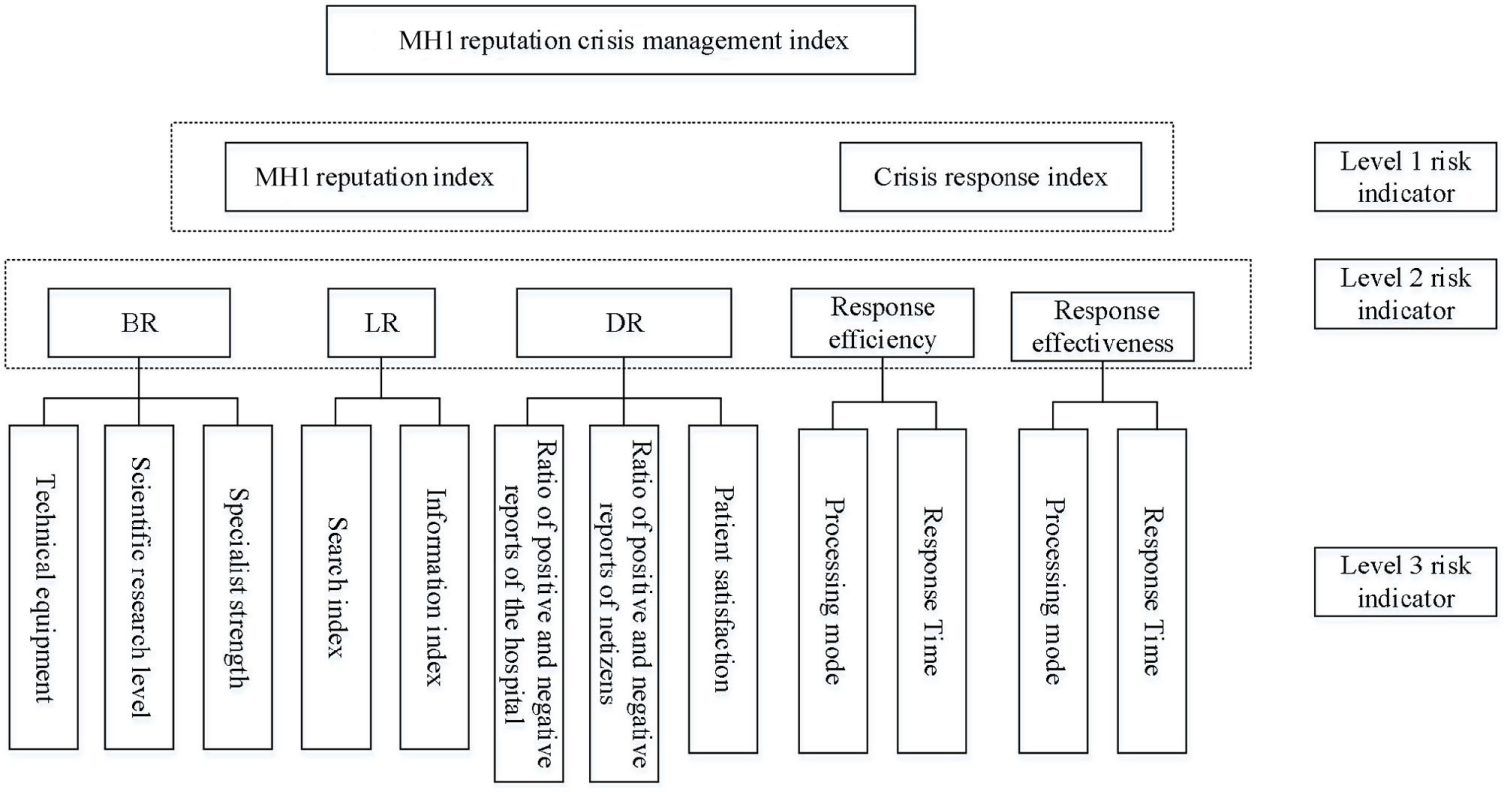
Architecture diagram of MHI reputation crisis management index.

The traditional reputation index is generally used to measure the overall reputation level of the target entity over a period of time, and its performance characteristics are relatively single. As a subject that is constantly developing and changing, a MHI reputation level will be affected by various crises inside and outside of the hospital. To expand the scope of research and show the MHI reputation crisis management level more three-dimensionally, this article starts from the two dimensions of “one static and one move” and combines the static reputation index with the dynamic crisis management level to comprehensively describe the MHI reputation management situation in a three-dimensional manner, as shown in Figure 1.

The MHI reputation crisis management index is composed of the MHI reputation index and the crisis response index. For the MHI reputation index, this paper evaluates the index through the comprehensive strength of the MHI itself and the evaluation of the MHI by the outsiders, namely the breadth of reputation (BR), strength of reputation (SR), and the degree of reputation (DR). On the other hand, for the crisis response index, this paper comprehensively considers the MHI's response efficiency and effectiveness when a reputation crisis occurs.

In the index model, the three dimensions of the MHI reputation index, namely the BR, SR, and DR, respectively, represent the popularity of the MHI, the strength of the MHI itself, and the evaluation obtained by the MHI, which can more comprehensively depict the reputation level. The SR takes into account the MHI technical equipment, scientific research level, and specialist strength and reflects the MHI own strength from the perspectives of hardware and software. The BR is constructed using the search index and information index in the *Baidu index* to reflect the influence scope of the MHI itself. The DR is based on the three subjects of MHI, netizens, and patients and comprehensively describes the MHI evaluation level from an objective perspective based on the ratio of positive and negative reports of the MHI, the ratio of positive and negative evaluations of netizens, and the satisfaction of patients. In addition, the crisis response index of the MHI includes crisis response efficiency and crisis response effect. The crisis response efficiency mainly depends on the MHI's handling methods and processing time. It is also a more conventional crisis response evaluation index.

### Sample Selection and Empirical Results

In this paper, the UH, TJ, ZN, RM, and MC were selected as the research objects. On the one hand, the research of the MHI reputation evaluation and management level needs to obtain the hardware and software of the MHI before and after the crisis, and the general MHI do not have perfect relevant information, that is, the lack of scientific processing or analysis, so its reference is limited. On the other hand, UH, TJ, ZN, RM, and MC are all well-known MHI in Hubei Province and even the whole country, with great actual influence, more representative and more research value.

Based on the above analysis, this paper collected and processed the data according to the construction model of MHI reputation crisis management index evaluated the specific crisis event indicators in each MHI, and finally calculated combined with these data. Among them, the data on the MHI technical equipment, scientific research level, and specialist strength are all from the relevant official websites, knowledge websites, major forums, and other public platforms. The search index and information index came from the *Baidu Index* from January 2020 to July 2021, and all kinds of emotional tendency data about the Internet platform are provided by the *Qingbo Public Opinion System*. In addition, before counting the data needed for the crisis response index, this article searched the crisis events of each MHI finally chose major events such as medical disturbance and medical accidents, and recorded the malignant degree of the crisis event and the MHI response, so as to complete the construction of the crisis response index. After obtaining data for all three indicators, the text is normalized, delegated and processed, and finally obtained from the bottom to top, as shown in Tables 1, 2.

**Table 1:** Score of each index of MHI reputation crisis management.

**Level 1 indicators**	**MHI score**					**Level 2 indicators**	**MHI score**				
	**TJ**	**UH**	**ZN**	**RM**	**MC**		**TJ**	**UH**	**ZN**	**RM**	**MC**
MHI reputation index	9.61	9.15	6.07	7.46	6.81	SR	8	8.48	6.88	7.46	5.62
						BR	10.54	11.02	10.01	9.7	7.22
						DR	9.55	8.78	4.26	6.65	7.21
Crisis response index	7	7.7	8.6	6.4	6.15	Response efficiency	9	9	9	6	8.5
						Response effectiveness	5.67	7.33	7.67	6.67	4.33

**Table 2:** MHI reputation crisis management index score.

**MHI reputation crisis management index**	**TJ**	**UH**	**ZN**	**RM**	**MC**
	8.7	8.65	6.95	7.09	6.58

It can be seen from Table 1 that the index model created in this paper is specific, among which the reputation strength and BR of UH are the highest, which not only is outstanding, but also has large enough influence to enjoy a high reputation in the industry. From the perspective of the media, netizens, and patients, TJ has a higher reputation; that is, it has received a higher evaluation. From the perspective of view of crisis response, the average response efficiency of TJ, UH, and ZN responded quickly and was handled properly in the face of crisis events. In comparison, RM and the MC are lacking. Due to the different MHI nature of the crisis is different, so even timely, handled properly which may not be able to calm the negative emotions. So it can be seen from Table 1, the UH, ZN, and RM's response effectiveness is higher, before and after the crisis treatment, people's emotional change is obvious. From the final reputation crisis management score, TJ and UH are both at a relatively high level, ZN and RM are close, and MC is the lowest. This also shows that TJ and UH have a better response mechanism for crisis management under their own good foundation, and for MC, their own strength will cause more crisis events; if the response mechanism is not reasonable, it will cause more serious consequences.

### MHI Reputation Evaluation Based on an Exponential Model

**Figure 2:**
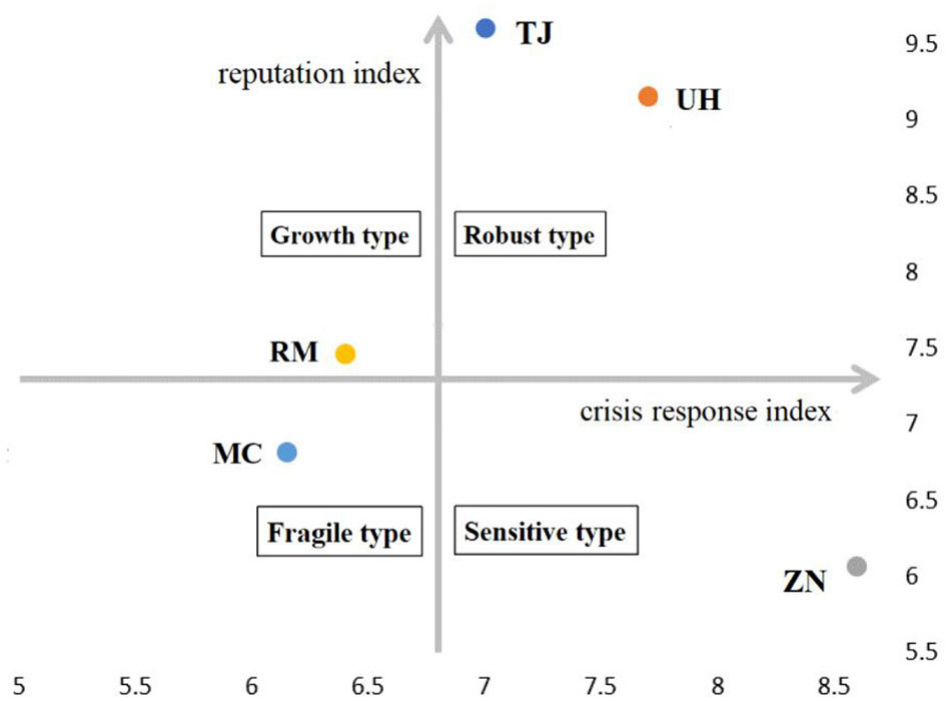
Coordinate chart of the MHI reputation crisis management index.

The above MHI reputation crisis management model divides the index into reputation index and crisis response index. This paper considers two indicators comprehensively, starting from the reputation level of the MHI itself and crisis response ability, so as to dynamically describe the overall reputation of the MHI. Therefore, this paper uses the MHI crisis response index as the horizontal axis and reputation index as the vertical axis to establish a coordinate chart as shown in Figure 2.

It can be seen from Figure 2, the TJ and UH are located in the first quadrant, namely “sensitive,” and the reputation index and crisis response index are very high, indicating that they are strong and capable of dealing with the crisis. RM is located in the second quadrant, namely the “growth type,” with a high reputation index, but a low crisis response index. When RM faces the crisis event and does not timely respond to the society, it is in a passive position, which leads to a long duration of public opinion and a large negative intensity, and its crisis response index is relatively low. The MC fell into the third quadrant, namely the “fragile type,” which mainly contains two aspects. On the one hand, its own structure is fragile and reflected in the MHI which has its own scientific research level, specialty strength, MHI construction and other aspects. On the other hand, it is more fragile to deal with crisis events; that is, the MHI does not respond in time or the crisis event is more malignant, and it is difficult to quell the online public opinion and eventually lead to the reputation damage. ZN located in the fourth sensitive quadrant is characterized by a low reputation index and a high crisis response index. From the specific situation of the five MHI, ZN has a relatively low sense of existence and low social reputation, which leads to a low reputation index. The crisis response index is timely responded and properly handled in case of crisis events, which shows that ZN has a strong ability to withstand pressure.

Based on the above analysis, it can be seen that after conducting reputation crisis management assessment, MHI managers need to clarify their own positioning, take different measures for different positionings, and achieve “grasp both hands and hard hands” in the reputation index and crisis response index, so as to effectively respond to external crises while improving their own reputation.

## Analysis and Countermeasures of MHI Reputation Crisis Management Based on SCCT

### Classification of MHI Reputation Crisis Based on SCCT

Situational crisis communication theory defines the crisis from the subjective perspective of the audience and judges, the responsibility of the event according to the attribution theory in psychology (19). Coombs divided crisis types into 10 categories as shown in Table 3 according to crisis ethnic group and degree of responsibility.

**Table 3:** Classification of crisis types in SCCT.

**Crisis group**	**Crisis type**	**Responsibility degree**
Victim crisis	Natural disaster	Minimal crisis responsibility
	Rumor	
	Emotional office abuse	
	Product tampering	
Accidental crisis	Query	Lower crisis responsibility
	Technical fault accident	
	Technical negligence for product damage	
Preventable crisis	Human negligence accident	Higher crisis responsibility
	Human negligence for product damage	
	Organizational misbehavior	

The 10 types of three clusters of Coombs are most widely used in crisis research. Therefore, on this basis, this paper further divides the MHI reputation crisis according to the attributes of the MHI and the needs of reputation management. According to the SCCT and the degree of MHI reputation crisis, the top 10 MHI reputation crisis types under the three crisis groups are obtained as shown in Table 4. The reputation of the MHI in the crisis group of victims is relatively passive, and the degree of responsibility and crisis is relatively small. Measures such as active response to clarification and even legal proceedings can repair the impact on reputation in a timely manner. The reputation of MHI in accidental crisis groups is affected due to objective factors or Internet public opinion. The crisis responsibility is moderate, and the coping strategies are more important for reputation restoration. MHI in the preventable crisis group are directly at fault, and improper response measures will result in a serious reputation crisis.

**Table 4:** Classification of MHI reputation crisis types.

**Crisis group**	**Crisis degree**	**Crisis type**	**Explanation**
Victim crisis	Smaller	Malicious medical disturbance	Malicious medical disturbance by patients and their families
		Internet rumor	Spreading false-negative information on the Internet
		Emotional office abuse	MHI staffs are treated unfairly
Accidental crisis	Medium	Query	Citizens questioned MHI charges, services, etc.
		Technical fault accident	A certain probability of medical accidents directly caused by equipment, technology, etc.
		Technical negligence for product damage	A certain probability of side effects such as surgery and medicine
Preventable crisis	Larger	Human negligence accident	Medical malpractice directly caused by man-made
		Human negligence for product damage	Injury caused by human negligence in medicine, services, etc.
		Misdiagnosis	Doctor's misdiagnosis and harm to patients
		Violation of medical ethics	Doctors abuse patients or privately receive gifts and other behaviors that violate medical ethics

### MHI Reputation Crisis Management Response Strategy

**Figure 3:**
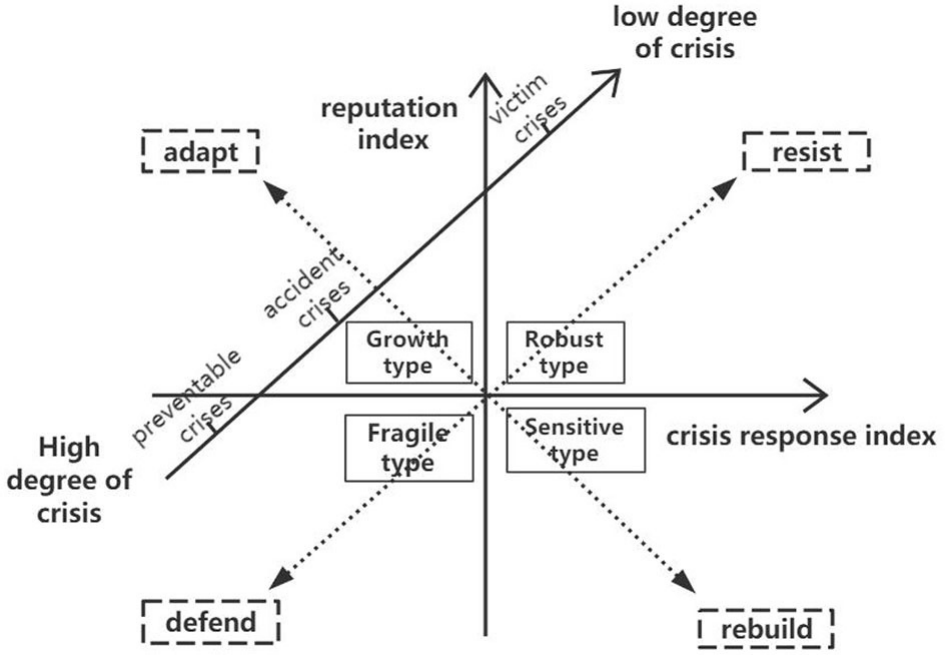
Distribution chart of MHI reputation crisis management response strategy.

According to the previous literature review and empirical analysis of the model, it can be found that due to the halo effect, MHI with different reputation bases are greatly affected when a crisis occurs. MHI with a high reputation crisis management index have a strong reputation foundation and crisis response ability and often are more vulnerable to public trust, and the threat to the MHI reputation under the same crisis type is low, so response strategies cannot be selected based on the type of crisis. Based on the three-cluster crisis response strategy of Coombs and the reputation crisis management foundation of MHI, the following crisis management response strategy is obtained in Figure 3.

Figure 3 is composed of MHI reputation positioning types, reputation management strategies, and crisis scenario types. The closer the MHI reputation position quantified by the MHI reputation crisis management index is to the upper right area, the more suitable it is for defensive response strategies. The closer you are to the lower left area, the more suitable the coping strategy for gentle defense. In the same way, the closer the type and DR crisis are to the upper right area, the more suitable it is for defensive response strategies, and the closer it is to the lower left area, the more suitable it is for a mild defense response strategy. In the context of crisis scenario communication theory, reputation management strategies dynamically match the type of MHI reputation positioning and crisis scenario types.

(1) For “robust” MHI with a strong reputation basis and crisis management, defensive management response strategies can be applied, such as handling rumors and malicious medical disturbances. through legal channels, and adopting open and tough measures for unexpected or preventable crises. Due to its good reputation, the public is more inclined to stand on the MHI's position and hold a trust attitude when facing negative information from MHI. At the same time, because of its rich experience in crisis management, MHI have the ability to control the negative impact of crisis events when adopting defensive response strategies.
(2) The location of the MHI reputation is close to the lower left area, indicating that the MHI reputation foundation and crisis management capabilities are weak and belong to “vulnerabilities.” Once a crisis occurs, it will have a greater impact on reputation. Therefore, a gentle defensive strategy should be used for crisis management to prevent negative information from spreading again, which exceeds the MHI's ability to respond to a reputation crisis.
(3) For “sensitive” MHI with low reputation index but high crisis response index, the “reconstruction” response strategy is applied; that is, private mediation plans such as compensation and apology are adopted when a crisis occurs, to avoid the spread of negative information, and once again attack the weak reputation foundation. At the same time, “sensitive” MHI should pay more attention to basic reputation management, enhance their reputation breadth, strength, and reputation, and rebuild public trust.
(4) For “growth” MHI with a high reputation index but a low crisis response index, adaptive management strategies should be used, and an appropriate crisis management plan should be selected according to the type of crisis and the degree of responsibility. “Growth” MHI have reputation-based advantages, but due to the lack of corresponding crisis management experience, directly adopting defensive response strategies may further create other reputational risks. Therefore, “growth” MHI gradually improve their reputation management capabilities in debugging response strategies and transform to a “robust” positioning.

In summary, from the SCCT perspective, the adapted response strategy refers to a crisis response strategy that matches the type of crisis. It can be obtained from the strategy distribution map that the victim's crisis risk is low, which can be applied to the countermeasure management strategy; the preventable crisis risk is high, and a gentle defensive strategy should be used.

### Five-Case MHI Reputation Management Strategy

In a case study of five MHI in Hubei Province, the reputation management strategy in Table obtained according to the one shown in Table 5.

1. TJ and UH are suitable for the strategy of “resist” reputation management.

**Table 5:** Five MHI reputation positioning and management strategies.

**Reputation positioning**	**MHI**	**Reputation crisis management index**	**Reputation management strategies**	
Robust type	TJ	8.70	Resist	
Robust type	UH	8.65	Resist	
Growth type	RM	7.09	Adopt	Resist (Victim crisis)
				Rebuild (Accidental crisis)
				Defend (Preventable crisis)
Fragile type	MC	6.58	Defend	
Sensitive type	ZN	6.95	Rebuild	

TJ and UH have obvious reputation management advantages among the five MHI as a whole, but they still need to improve their ability to respond to reputation crises. TJ and UH are robust types of MHI reputation positioning, and their BR and SR have absolute advantages. Therefore, in the face of negative public opinions such as “difficulty in registering” and “unreasonable fees,” they should adopt active and defensive management strategies, dealing with malicious and false information through legal channels, responding to patient complaints in a timely manner, opening official accounts in major medical-related forums, paying attention to negative information, and actively interacting with netizens. In the process of reputation crisis management, TJ and UN can pay more attention to the prevention of socially sensitive events and deal with them in advance through crisis management cases in other MHI.

2. RM is suitable for the strategy of “adopt” reputation management.

RM is positioned as a growth-oriented reputation. It has certain reputation-based advantages but weak crisis response capabilities. It is suitable for adaptive management strategies; that is, when a reputation crisis occurs, first evaluate the risk status and learn from other MHI solutions to build reputation to manage the case database and gradually explore the reputation crisis management plan that suits the positioning and style of the MHI. RM should strengthen the monitoring of reputation crisis events and try to intervene in advance when the crisis expands.

3. MC is suitable for the strategy of “defend” reputation management.

MC is inferior to other MHI in terms of its own strength, such as BR and SR, and it has insufficient experience in handling reputation crises. It is in a fragile reputation position, so it is suitable for a moderate defense management strategy; first, pay attention to the internal management of MHI, strengthen the standardization of MHI staff training and product use, and minimize the probability of preventable crises; second, more public welfare activities can be carried out in the community and the Internet to gradually increase BR and DR.

4. ZN is suitable for the strategy of “rebuild” reputation management.

ZN has a low reputation index and is in a sensitive reputation position. It needs to rebuild its management strategy for reputation restoration. ZN has the strongest crisis management capabilities among the five MHI, but has the lowest DR. Therefore, on the one hand, it should maintain a high degree of sensitivity to negative information on the Internet and respond in a timely manner; on the other hand, it should rectify the content of patient complaints; in the event of an incident, try to choose a private mediation method to avoid secondary negative information due to the weak reputation at this stage.

## Discussion and Conclusion

This paper constructs a MHI reputation crisis management index model and divides the index into two indexes, namely, a static MHI reputation index and a relatively dynamic crisis response index. The data of the five MHI UH, TJ, ZN, RM, and MC are used to compare the two indexes. These indicators were further subdivided and quantified, and finally the reputation crisis management index of these five MHI was evaluated and analyzed. The analysis results show that different MHI have their own characteristics in the two dimensions of reputation index and crisis response index. This article divides them into four types. Among them, TJ and UH are classified as “robust type” because of the high two indexes. The crisis response index of RM is low and is classified as “growth type.” The two indicators of the MC are low and are classified as “fragile type.” ZN has a low reputation index and is classified as “sensitive type.”

Second, according to the three-cluster ten-type crisis research of Coombs, this paper classifies the MHI reputation crisis according to SCCT and the degree of MHI reputation crisis and redefines the ten types of MHI reputation crisis from the three degrees of victim crisis, accidental crisis, and preventable crisis.

Finally, based on the above theories, this paper analyzes the targeted crisis response strategies of five sample MHI. Among them, the “robust type” MHI, namely TJ and UH, are suitable for the countermeasures of defensive management. “Fragile type” MHI, namely the MC, are suitable for mild defense strategies. “Sensitive type” MHI, namely ZN, should adopt a “rebuild” strategy, that is, promptly communicate compensation and apologies after a crisis event to avoid further expansion of the spread of negative information. “Growth type” MHI, namely the RM, should choose appropriate response strategies based on the degree of crisis and the degree of responsibility and require more flexibility.

## Data Availability Statement

The original contributions presented in the study are included in the article/supplementary material, further inquiries can be directed to the corresponding authors.

## Author Contributions

YL: writing—original draft preparation and conceptualization. XL: designing the framework and data collection. RD: methodology. TH: software. X-jW: writing—reviewing and editing. All authors contributed to the article and approved the submitted version.

## Funding

This work was supported by the Philosophy and Social Science research Project in Department of Education of Hubei Province (Grant no. 21G001), the National Social Science Fund of China (Grant no. 20&ZD058), and the Construction of Science and Technology Planning Project of Hubei Province in 2020 (Grant no. 2020041).

## Conflict of Interest

XL was employed by company Hubei Media Group, Wuhan. The remaining authors declare that the research was conducted in the absence of any commercial or financial relationships that could be construed as a potential conflict of interest.

## Publisher's Note

All claims expressed in this article are solely those of the authors and do not necessarily represent those of their affiliated organizations, or those of the publisher, the editors and the reviewers. Any product that may be evaluated in this article, or claim that may be made by its manufacturer, is not guaranteed or endorsed by the publisher.

## Abbreviations

MHI: Medical and health institutions
UH: Union Hospital Tongji Medical College Huazhong University of Science and Technology
TJ: Tongji Hospital, Tongji Medical College Huazhong University of Science and Technology
ZN: Zhongnan Hospital of Wuhan University
RM: Renmin Hospital of Wuhan University
MC: Maternal and Child Hospital of Hubei Province
SCCT: Situational crisis communication theory
BR: Breadth of reputation
SR: Strength of reputation
DR: Degree of reputation.
